# Two novel anticancer compounds with minimum cardiotoxic property

**DOI:** 10.1186/s40360-020-00457-8

**Published:** 2020-11-19

**Authors:** Tayebeh Afsharirad, Raheleh Tahmasvand, Mohsen Amini, Bahram Daraei, Mona Salimi

**Affiliations:** 1 Department of Toxicology, Faculty of Medical Sciences, Tarbiat Modares University, Tehran, Iran; 2 Physiology and Pharmacology Department, Pasteur Institute of Iran, P.O. Box 13164, Tehran, Iran; 3 Department of Medicinal Chemistry, Faculty of Pharmacy, Tehran University of Medical Sciences, Teharn, Iran

**Keywords:** Cardiotoxicity, Apoptosis, ROS, Mitochondrial potential

## Abstract

**Background:**

Although two novel synthesized compounds with tri-aryl structures; 3-(4-chlorophenyl)-5-(4-fluorophenyl)-4-phenyl-4,5-dihydro-1,2,4-oxadiazole (**A**) and 3,5-bis-(4-chlorophenyl)-4-phenyl-4,5-dihydro-1,2,4-oxadiazole (**B**) have been previously demonstrated to possess remarkable anti-breast cancer activity, their cardiotoxicity remains a major concern due to their mechanism of action. To address this concern, we assessed the ability of these compounds to cause toxicity towards H9c2 cardiomyocytes as an in vitro model of cardiotoxicity.

**Methods:**

Cytotoxic activity of both compounds was explored in vitro on H9c2 cells using MTT assay. Annexin V/PI method, intracellular ROS determination and mitochondrial membrane potential assay were applied to elucidate the mechanism of action of the cell death.

**Results:**

MTT assay revealed a concentration- and time-dependent cardiotoxicity. Findings of apoptosis by double staining with annexin V and propidium iodide divulged no cell death including apoptosis and necrosis at the concentration that were effective to inhibit cancer cells proliferation (10 μM) at 24 and 48 h. Furthermore, flow cytometric measurement of membrane potential and ROS determination using DCFH-DA verified the safe concentration of the compounds against H9c2 cells with no cardiotoxic effect. However, the higher concentration of the compounds could induce cell death through ROS-mediated mitochondrial dysfunction.

**Conclusions:**

Altogether, the results represented two novel chemical molecules possessing anti-breast cancer activity with minimum cardiac side effect.

## Background

Cardiovascular diseases (CVDs) remains number one of the fatal diseases, which is ever increasing worldwide and hence, scientific community has concerned about it [1, 2]. Cardiotoxicity occurs as a result of the complicated working of cardiac cells and tissues or due to a chemical molecule affecting heart function and structure [3, 4]. Cardiotoxicity induced by drugs has gained attention in the past few decades. In this context, anticancer therapy has a direct impact on cardiac function and the most common toxicities of heart are due to cancer treatment [5]. However, clinicians still use many cardiotoxic drugs due to their beneficial effects, which outweigh cardiac malformations risks [4]. As it was above-mentioned, most of the anticancer drugs exhibit a wide range of cardiovascular toxicities which results in stopping cancer treatment and affects the short- and long-term quality of life [6–8].

Inflammation and oxidative stress are mutually reliant processes, which predominantly involve in cardiovascular diseases and cancers via inducing apoptosis and necrosis [9–13]. In other words, high level of reactive oxygen species (ROS) due to oxidative stress can enfeeble cardiac cellular signaling pathways [14].

**Fig. 1:**
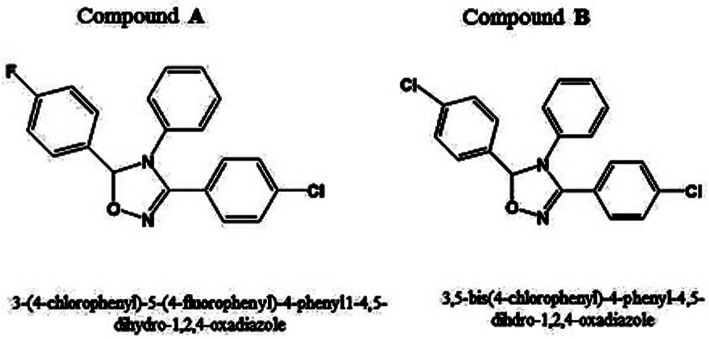
Chemical structures of compounds **A** and **B**

Triaryl template-based structures found in a wide array of compounds, display various biological properties including anticancer [15, 16]. Besides it, a number of compounds with this pivotal structural feature revealed cyclooxygenase-2 (COX-2) inhibitory activity [17]. In this line, we previously reported the apoptotic activity of the two compounds; 3-(4-chlorophenyl)-5-(4-fluorophenyl)-4-phenyl-4,5-dihydro-1,2,4-oxadiazole (**A**) and 3,5-bis-(4-chlorophenyl)-4-phenyl-4,5-dihydro-1,2,4-oxadiazole (**B**) (Fig. 1) in the breast cancer cells via a COX-2 independent pathway [18], although these compounds interacted with the active site of COX-2 enzyme [19]. Notably, different hypothesis were raised by researchers for the discernible increase in cardiotoxicity due to apoptosis in the terminally differentiated cardiomyocytes [20]. Interestingly, amongst different pathophysiological consequences, cardiovascular diseases are associated with apoptosis. Indeed, a number of studies provided convincing evidence that cardiac cell death has been contributed to major heart diseases including myocardial infarction (MI), cardiomyopathies, arrhythmogenic right ventricular dysplasia, end-stage heart failure, etc. [21–23].

Given the importance of cardiac cell apoptosis and knowing that major mechanism related to the two tested compounds is triggering mitochondrial apoptosis, motivated us to evaluate the cardiotoxicity of the compounds. Since generation of ROS activates mitochondrial-mediated apoptotic signaling pathway leading to caspase 3 activation and cardiac cell apoptosis, we further focused on the understanding of molecular mechanism underlying cardiotoxicity of two compounds. To achieve it, a cell culture model created by rat cardiomyoblast cell line (H9c2) was applied.

## Materials and methods

### Cell culture

H9c2 (Rat embryonic cardiac myoblast) cell line (C585) was obtained from National Cell Bank of Pasteur Institute of Iran. The cells were cultured in Dulbecco’s Modified Eagle’s Medium (DMEM) (Gibco-BRL, Rockville, IN) containing high level of glucose and 2 mM L-glutamine supplemented with penicillin-streptomycin (Gibco-BRL, Rockville, IN) and Fetal Bovine Serum (FBS) (Gibco-BRL, Rockville, IN) at 37 °C in a humidified incubator with 5% CO2. Compounds **A** and **B** were reconstituted in Dimethyl Sulfoxide (DMSO) and the final concentration of DMSO was 0.5%. Compounds **A** and **B** were synthesized by the Medicinal Chemistry Research Laboratory at the Faculty of Pharmacy, Tehran University of Medical Sciences. Cells without treatment were considered as negative control.

### MTT assay

Cell viability was determined by MTT assay. H9c2 cells were plated in 96-well plates at 7000 cells/well for 24 h and 5000 cells/well for 48 h of treatment. Cells were exposed to different concentrations of compounds **A** and **B** to reach final concentration of 0.1–100 μM at 24 and 48 h. Afterwards, 20 μl of MTT (3-(4,5-dimethylthiazol-2-yl)-2,5-diphenyl tetrazolium bromide,5 mg/ml) (Sigma-Aldrich, Saint Louis, Missouri, USA) was added for an additional 4 h. The medium was removed and 200 μl of DMSO was added to each well to dissolve formazan crystals. Finally, the absorbance was measured by microplate reader (Epoch2, Biotek, USA) at 545 nm.

### Intracellular ROS determination

To determine the intracellular ROS, H9c2 cells were seeded in 6-well plates at a concentration of 25 × 10^4^ cells per well for 24 h and 15 × 10^4^ cells for 48 h of incubation. Cells were then treated with 10 and 50 μM of compounds **A** and **B** for 24 and 48 h. Following incubation of cells with 10 μM of DCFH (Sigma-Aldrich, Saint Louis, Missouri, USA) at 37 °C for 20 min, the cells were washed with PBS (Phosphate Buffered Saline) and then harvested to detect ROS level by flow cytometer.

### Mitochondrial membrane potential assay

Mitochondrial dysfunction was assessed using mitochondrial membrane potential (ΔΨ m) assay. Briefly, H9c2 cells were plated in 6-well plates as described above and then trypsinized after treating with 10 and 50 μM of compounds **A** and **B** for 24 and 48 h. We used carbonyl 3-chlorophenylhydrazone (CCCP) as a standard control. After washing the cells with PBS, 0.5 ml of PBS buffer containing 10 μg/ml of JC-1 (Mitoprobe JC1 assay kit, Life technologies, USA, M34152) was added to the suspend cells. Following 15–30 min of incubation at 37 °C, cells were centrifuged to discard the supernatant. Cell pellets were then re-suspended in PBS and analyzed by flow cytometry. A mitochondria-sensitive dye (JC-1) was used to monitor mitochondrial membrane potential alterations by observing the percentage of mitochondrial matrix aggregation. The aggregate JC-1 (red fluorescence) was determined at the emission wavelength of 590 nm, and the monomeric JC-1 (green fluorescence) monitored at 529 nm.

### Apoptosis assay

The numbers of apoptotic cells were measured by AnnexinV/PI assay kit (Roche Applied Science, Indianapolis, IN, USA) according to the manufacture’s instruction. To do it, H9c2 cells were washed and then harvested after treatment with 10 and 50 μM of compounds **A** and **B**. Doxorubicin was used as a positive control. Next, cells were stained using 100 μl of Annexin V-FLUOS labeling solution containing 2 μl annexin V-FLUOS labeling agent, 2 μl PI solution and 1 ml incubation buffer. Cells were then incubated for 15 min at 37 °C and subjected to flow cytometry. The percentages of viable (AnnexinV−/PI−), early apoptotic (AnnexinV+/PI−), late apoptotic (AnnexinV+/PI+) and necrotic (AnnexinV−/PI +) cells were finally analyzed.

### Statistical analysis

At least three biological replicates were considered for all experiments and the data were expressed as mean ± SEM. To compare the differences between multiple parameters, one-way ANOVA followed by the Tukey’s post test was applied using GraphPad Prism 6.0 Software. A *p* value lower than 0.05 was considered as significant.

## Results

### Compounds A and B affected viability of H9c2 cells

**Fig. 2:**
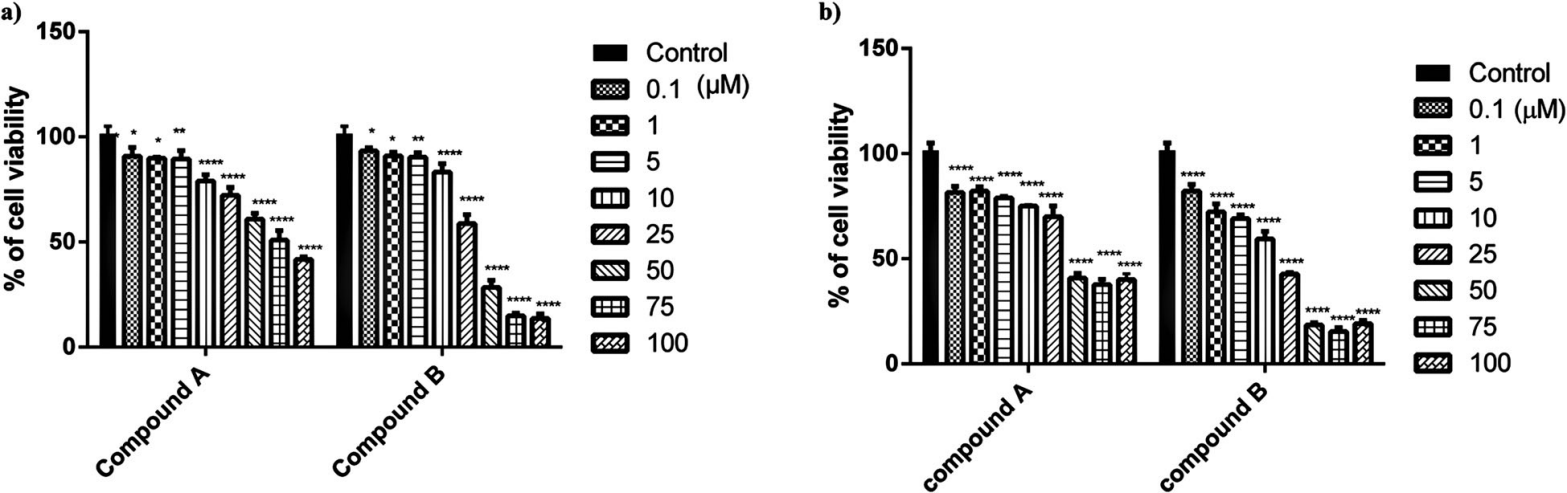
Concentration-dependent cytotoxicity of compounds **A** and **B** on H9c2 cells at (**a**) 24 h and (**b**) 48 h. Values are presented as mean ± SEM of three independent experiments, performed in triplicate.^*^p < 0.05,^**^p < 0.01,^***^p < 0.001,^****^p < 0.0001 compared with control

H9c2 cells were treated with different concentrations of compounds **A** and **B** for 24 and 48 h and cell viability was determined using MTT assay. As demonstrated in Fig. 2, both compounds reduced the cell viability in a time- and concentration-dependent manner within the concentration range of 0.1–100 μM. 75 and 25 μM concentrations of compound **A** reached the myocardial viability cells to 50.9 and 72.2%, respectively, after 24 h. These values for compound **B** were 14.9 and 58.7%, which were close to their IC_50_ concentrations (IC_50_ **A**: 77.02 ± 1.09 and IC_50_ **B**: 28.64 ± 1.04 μM). The vitality of the cells was also diminished more significantly after 48 h of treatment compared with that of 24 h (IC_50–48 h_ was 45.90 ± 1.17 μM for compound **A** vs. 10.68 ± 1.15 μM for compound **B**). Considering our previous findings implying the effective concentration of both compounds on breast cancer cells (~ 10 μM) and that this concentration is below the IC_50_ values in myocardial cells, we selected 10 μM for use in our following experiments. Moreover, we chose a 5-fold IC_50_ concentration to verify our results.

### Apoptosis induction by compounds A and B in H9c2 cells

**Fig. 3:**
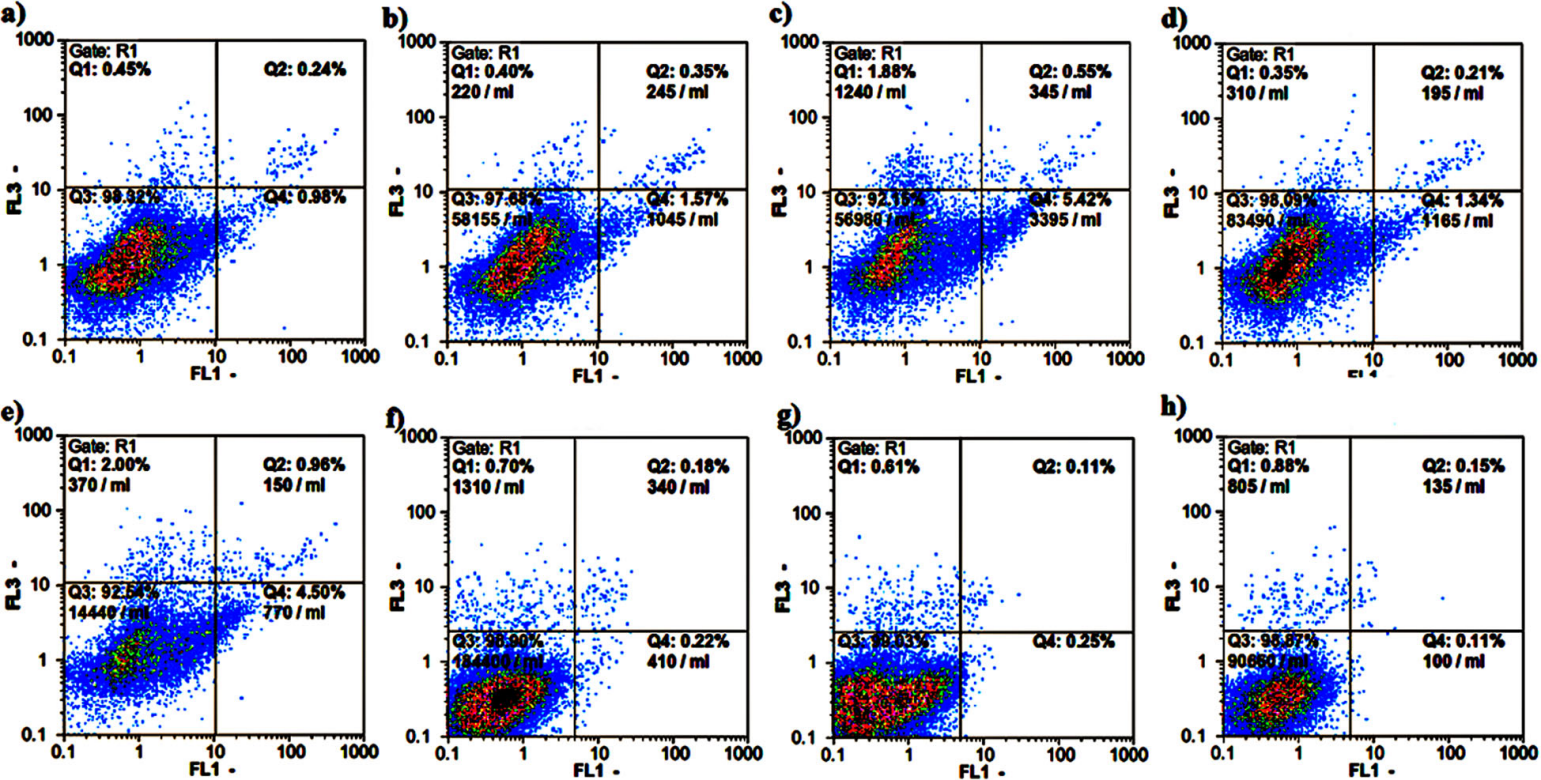
Annexin-V/PI flow cytometric analysis to quantify apoptosis induction in H9c2 cells. Dot plot of (**a**) H9c2 cells alone and treated with compound **A** at (**b**) 10 μM, (**c**) 50 μM and compound **B** at (**d**) 10 μM, (**e**) 50 μM for 24 h; (**f**) H9c2 cells alone and treated with compounds (**g**) **A** and (**h**) **B** at 10 μM for 48 h The results shown are representatives of three independent experiments. Quadrant 3, living cells An−/PI−; Quadrant 4, early apoptotic cells An+/PI−; Quadrant 2, late apoptotic cells An+/PI+; Quadrant 1, necrotic cells An−/ PI−

Flow cytometry was applied to investigate apoptosis in the control and the compounds-treated H9c2 cells. Cell apoptosis was determined using Annexin V-FLUOS/Propidium iodide (PI) double staining. The apoptotic rate was concentration -dependently increased in the H9c2 cells treated with both compounds at 24 h compared with the control group (Fig. 3a-e). In this context, 10 μM of compounds **A** and **B** did not affect the cell, while a large proportion of cells were in the early apoptotic stage when the cells were subjected to both compounds at 50 μM (Table 1). Of note, no apoptotic cells were seen upon treatment of H9c2 cells with 10 μM of compounds **A** and **B** at 48 h (Fig. 3f-h and Table 1). Due to the highest rate of cell death following treating with 50 μM of the compounds at 48 h, we excluded it and focused our further experiments on 10 μM.

**Table 1:**
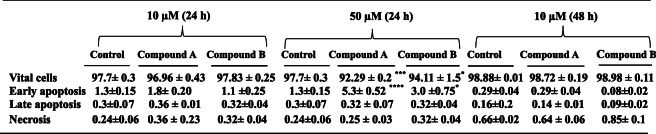
Percentage of H9C2 cells in each state after treatment with two concentrations of compounds **A** and **B** at 24 and 48h^**a**^

^a^The data presented are the mean ± SEM of three independent experiments. ^*^p < 0.05,^**^p < 0.01,^***^p < 0.001,^****^P < 0.0001 compared with control

### Apoptosis induced by compounds A and B is through ROS generation

**Fig. 4:**
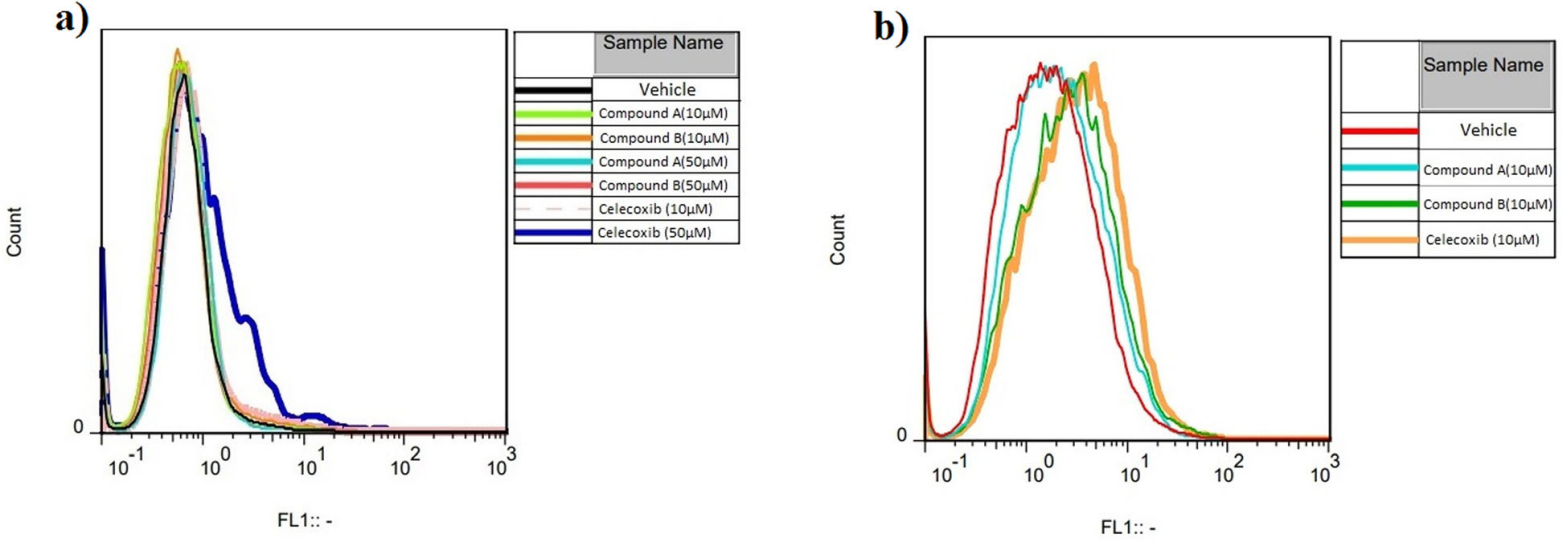
ROS generation in H9c2 cells after (**a**) 24 h and (**b**) 48 h. ROS was measured in the untreated H9c2 cells (control/vehicle) group as well as the cells treated with compounds **A** and **B** at 10 and 50 μM

Previous studies have revealed that cell apoptosis can occur as a result of ROS generation through regulation of caspase [24]. To explore whether compounds **A** and **B** affect ROS generation at 10 and 50 μM concentrations after 24 and 48 h of incubation, we used DCFH-DA to determine intracellular ROS level in H9c2 cells. DCFH-DA freely enters the cell and is oxidized to DCF by ROS inside the cell which produces green fluorescence signals. As presented in Fig. 4a, no significant shift was seen after 24 h of treatment of both compounds at 10 and 50 μM compared with the control group. However, an observable shift of fluorescence was detected in H9c2 cells treated with 10 μM of compounds **A** and **B** at 48 h (Fig. 4b).

### Compounds A and B influenced mitochondrial potential membrane

**Fig. 5:**
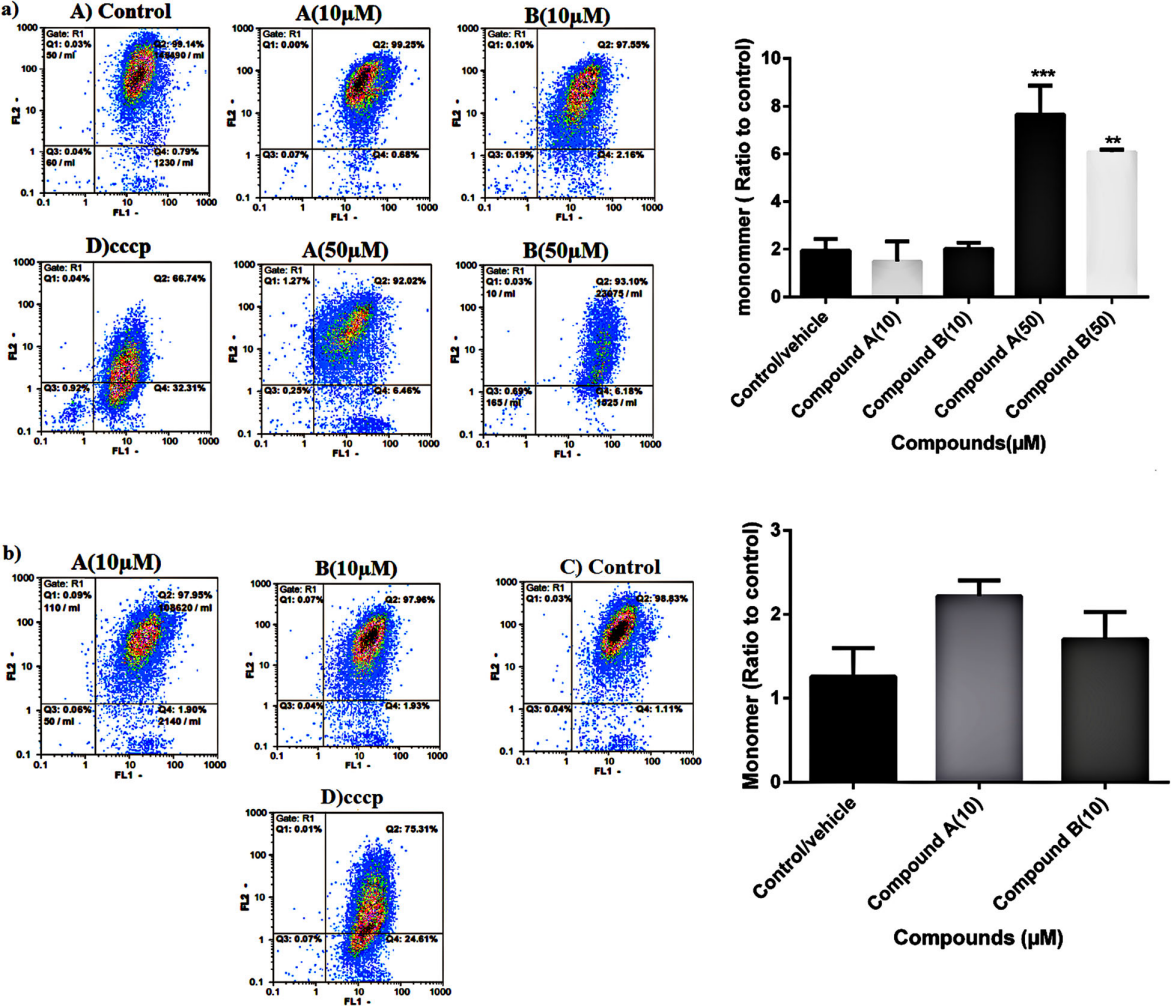
**a** Destabilization of the mitochondrial membrane of H9c2 Cells. H9c2 cells were treated for 24 h with compounds **A** and **B** at 10 and 50 µM, following which, they were incubated with JC-1 mitochondrial membrane permeable dye. Fluorescence readings were obtained using image based flow cytometry; **b**. H9c2 cells were treated for 48 h with compounds **A** and **B** at 10 µM. Analyses were performed on 3 independent experiments and values are expressed as mean±SEM. **p < 0.01, ***p < 0.001 versus vehicle/ control

Since mitochondrial function is highly sensitive to oxidative damages [25] and to further verify whether mitochondrial apoptosis pathway is induced by compounds **A** and **B**, the mitochondrial membrane potential (MMP) was assessed using the fluorescent dye JC-1. Red fluorescence displaying the matrix of actively respiring mitochondria is the aggregate form of JC-1 molecules, while green fluorescence is emitted from the monomeric form of the JC-1 molecules indicating mitochondrial membrane depolarization. As shown in Fig. 5a, after treating with compounds **A** and **B** at 50 μM for 24, the percentage of JC-1 monomeric cells was increased compared with control, which is an indicator of the loss of MPP revealing the mitochondrial damage. No significant change was detected upon incubation of the cells with 10 μM of compounds **A** and **B** for 24 h. Similarly, no mitochondrial dysfunction was observed following treatment of the cells with both compounds at 10 μM for 48 h (Fig. 5b).

## Discussion

Evidences display that oxidative stress induces a variety of cardiomyocyte deaths, consisting of apoptosis, necrosis and necroptosis [26–28]. In this context, ample researches have shown that the therapeutic agents generating ROS cause cardiotoxicity [29]. Among different types of therapeutics, certain classes of chemotherapeutic agents induce more common and frequent cardiotoxic effects. On the other hand, heart dysfunction along with the promotion of cardiac hypertrophy and the loss of contractile occur due to apoptosis of cardiomyocyte, which in turn develops cardiovascular diseases [30, 31]. The anti-breast cancer activities of two novel synthesized compounds (**A** and **B)** have been previously studied [18]. Our findings revealed that these two compounds act through inducing apoptosis. Keeping in mind that apoptosis plays an important role in cardiotoxicity and in consequence heart dysfunction and knowing that these two compounds likely resemble to COX-2 inhibitors with tri-aryl structures exerting their effects via apoptosis induction [19], herein, we attempted to verify that these compounds are nontoxic to cardiac cells. Hence, we performed the in vitro experiments on H9c2 cells. This is the first study to find out the effectiveness of compounds **A** and **B** to preserve the cardiomyocyte viability.

In the current study, for an exposure time of 24 h, compounds **A** and **B** concentration-dependently decreased H9c2 cell viability. For longer period of exposure (48 h), these compounds also caused a decrease in cell viability implying a time –dependent cell cytotoxicity. Furthermore, our findings exhibited that the higher dose of compounds **A** and **B** (50 μM) can trigger the apoptotic process in H9c2 cells upon 24 h of treatment in which early apoptosis to a large extent was involved in the cell death. Interestingly, a concentration of 10 μM by which a remarkable anticancer activity had been seen for the compounds against breast cancer cells [18], could activate no apoptotic process at 24 and even 48 h on cardiac cells.

A large number of studies have elucidated that intracellular ROS generation is closely associated with cell apoptosis [32]. Consistent with the apoptosis data, our results from determining the intracellular ROS level revealed no shift in the fluorescent intensity in H9c2 cells after cell exposure to 10 μM of both compounds for 24 h. Surprisingly, when the cells were subjected to 50 μM of compounds **A** and **B** for 24 h, the level of ROS remained unchanged in H9c2 cells which can be explained regarding the time that the ROS level was determined in the treated cells. Indeed, we assumed that following an initial increase in ROS level, it reaches the original level after 24 h. Thus, a large population of the cells was in the early of apoptosis at 24 h, while no significant difference in ROS concentration was detected in the treated cells compared with the control. Moreover, after incubation of the cells with 10 μM of compounds **A** and **B** for 48 h, no apoptosis process was induced, whereas a small level of ROS was produced suggesting the triggering of oxidative stress which was not strong enough to induce cell apoptosis. Our data support previous findings and indicated that ROS contributes to the regulation of apoptosis but it is generated in two steps; early and late stages in which time of ROS determination plays a key role [33].

Deregulation of the different cell death pathways causes pathological outcomes for oxidative stress-associated diseases including ischemia-reperfusion injury that has to be considered as adverse effects of drugs [34]. The generated ROS can inaugurate and enhance important mitochondrial alterations by depolarizing MMP. In other words, ROS mainly causes depolarization and bulging of the mitochondria and then augments apoptotic mechanism through mitochondrial involvement [35, 36]. Thus, as a consequence, cytochrome *c* that is a key mediator in the mitochondrial pathway of cell death is released from mitochondria [37]. At last, upon activation of caspase-3, cell apoptosis is induced [38]. Our results demonstrated that 50 μM of compounds **A** and **B** disturbed mitochondrial membrane potential ψm of H9c2 at 24 h, whereas no mitochondrial function impairment was observed following treatment of H9c2 with 10 μM of both compounds for 24 h confirming our apoptosis results. Interestingly, it was shown that excessive ROS can damage mitochondria as well as open its permeability transition pore (PTP), and thereby induces mitochondrial permeability transition. The mitochondrial depolarization and outer membrane rupture due to these alterations result in cell apoptosis or death [39, 40]. These findings corroborate our obtained results and indicate that low dose of both compounds can induce ROS production after 48 h of treatment; however, this level of ROS was unable to induce apoptosis. Interestingly, high dose of these compounds after 24 h damaged mitochondrial potential and induced apoptosis, although it did not affected ROS generation suggesting an undetectable level of ROS due to the inappropriate time of measurement in the cells exposed to a high concentration of the compounds.

## Conclusions

Our study represented two novel chemical molecules possessing anti-breast cancer activity with minimum influence on the oxidative stress-mediated apoptosis induction on cardiomyocytes implying therapeutic potential for heart disease. However, these compounds at the higher dose can attenuate mitochondrial dysfunction and trigger apoptosis via oxidative stress. But, the detailed mechanism of action needing to be further investigated.

## Data Availability

The datasets used during the present study are available from the corresponding author on reasonable request.

## Abbreviations

CVDs: Cardiovascular diseases
ROS: Reactive oxygen species
COX-2: Cyclooxygenase-2
MI: Myocardial infarction
DMEM: Dulbecco’s modified Eagle’s medium
FBS: Fetal bovine serum
DMSO: Dimethyl sulfoxide
MTT: (3-(4,5-dimethylthiazol-2-yl)-2,5-diphenyl tetrazolium bromide
DCFH: 2′,7′-Dichlorofluorescin
PBS: Phosphate buffered saline
CCCP: Carbonyl 3-chlorophenylhydrazone
PI: Propidium iodide
SEM: Standard error of the mean
ANOVA: Analysis of variance
IC_50_: The half maximal inhibitory concentration
DCFH-DA: 2′,7′-Dichlorofluorescin diacetate
MMP: Mitochondrial membrane potential
PTP: Permeability transition pore
